# Case report: Metastatic urothelial cancer with an exceptional response to immunotherapy and comprehensive understanding of the tumor and the tumor microenvironment

**DOI:** 10.3389/fonc.2022.1006017

**Published:** 2022-10-31

**Authors:** Cora N. Sternberg, Nara Shin, Konstantin Chernyshov, Fabio Calabro, Linda Cerbone, Giuseppe Procopio, Natalia Miheecheva, Georgy Sagaradze, Alisa Zaichikova, Naira Samarina, Alexandra Boyko, Jessica H. Brown, Leysan Yunusova, Daniela Guevara, Jyothi Manohar, Michael Sigouros, Majd Al Assaad, Olivier Elemento, Juan Miguel Mosquera

**Affiliations:** ^1^ Englander Institute for Precision Medicine, Sandra and Edward Meyer Cancer Center, Weill Cornell Medicine, Hematology and Oncology, New York-Presbyterian, New York, NY, United States; ^2^ BostonGene, Corp, Waltham, MA, United States; ^3^ Special Operative Unite (UOS) Oncologia Tumori Genito-urinari, Department of Medical Oncology, San Camillo Forlanini Hospital, Rome, Italy; ^4^ Istituto Tumori, Department of Medical Oncology, Milan, Italy; ^5^ Englander Institute for Precision Medicine, Weill Cornell Medicine, New York, NY, United States; ^6^ Department of Pathology and Laboratory Medicine, Englander Institute for Precision Medicine, Weill Cornell Medicine, New York, NY, United States; ^7^ Englander Institute for Precision Medicine, Institute for Computational Biomedicine, Weill Cornell Medicine, New York, NY, United States

**Keywords:** urothelial cancer, bladder cancer, pembrolizumab, KEYNOTE-045 trial, tumor microenvironment, whole-exome sequencing, RNA sequencing, case report

## Abstract

Although immune checkpoint inhibitors (ICIs) are increasingly used as second-line treatments for urothelial cancer (UC), only a small proportion of patients respond. Therefore, understanding the mechanisms of response to ICIs is critical to improve clinical outcomes for UC patients. The tumor microenvironment (TME) is recognized as a key player in tumor progression and the response to certain anti-cancer treatments. This study aims to investigate the mechanism of response using integrated genomic and transcriptomic profiling of a UC patient who was part of the KEYNOTE-045 trial and showed an exceptional response to pembrolizumab. Diagnosed in 2014 and receiving first-line chemotherapy without success, the patient took part in the KEYNOTE-045 trial for 2 years. She showed dramatic improvement and has now been free of disease for over 6 years. Recently described by Bagaev et al., the Molecular Functional (MF) Portrait was utilized to dissect genomic and transcriptomic features of the patient’s tumor and TME. The patient’s tumor was characterized as Immune Desert, which is suggestive of a non-inflamed microenvironment. Integrated whole-exome sequencing (WES) and RNA sequencing (RNA-seq) analysis identified an ATM mutation and high TMB level (33.9 mut/mb), which are both positive biomarkers for ICI response. Analysis further revealed the presence of the APOBEC complex, indicating the potential for use of APOBEC signatures as predictive biomarkers for immunotherapy response. Overall, comprehensive characterization of the patient’s tumor and TME with the MF Portrait revealed important insights that could potentially be hypothesis generating to identify clinically useful biomarkers and improve treatment for UC patients.

## Introduction

The current first-line standard-of-care treatment for patients with metastatic urothelial cancer (UC) is platinum-based combination chemotherapy, with disease control occurring in 65% to 85% of patients. However, progression-free survival (PFS) and overall survival (OS) are often limited, as a median survival rate of 9 to 14 months is seen in patients who receive first-line platinum-based chemotherapy (1). Although methotrexate, vinblastine, doxorubicin, cisplatin (M-VAC) chemotherapy was developed in 1989 (1), few achievements have occurred in the past 30 years aside from gemcitabine (GC) and high-dose M-VAC (HD M-VAC) (2, 3). Until recently, only 25% to 55% of patients received second-line treatment with less than optimal results. Five immunotherapeutic agents have been approved as systemic therapy for UC, including atezolizumab, durvalumab, avelumab, nivolumab, and pembrolizumab. A phase III study (KEYNOTE-045 trial) showed that compared to standard-of-care chemotherapy, pembrolizumab led to a 27% OS benefit after platinum-based chemotherapy (4). Recent studies further demonstrated the potential for the use of immunotherapeutic agents in the maintenance setting for patients who respond to chemotherapy (5).

As the utilization of immune checkpoint inhibitors (ICIs) continues to increase for UC, comprehensive characterization of the tumor and tumor microenvironment (TME) is critical to understand the molecular mechanisms responsible for ICI response, which can then lead to the development of clinically useful biomarkers that can determine the most effective treatment for a patient (6–8). Bagaev et al. previously reported the development and utilization of a Molecular Functional (MF) Portrait to dissect genomic and transcriptomic features of tumors, including bladder cancer (9). Herein, we report a case of an exceptional response to pembrolizumab in the context of the KEYNOTE-045 study and utilization of the MF Portrait (9) to understand the underlying mechanisms of treatment response.

## Case history

**Figure 1:**
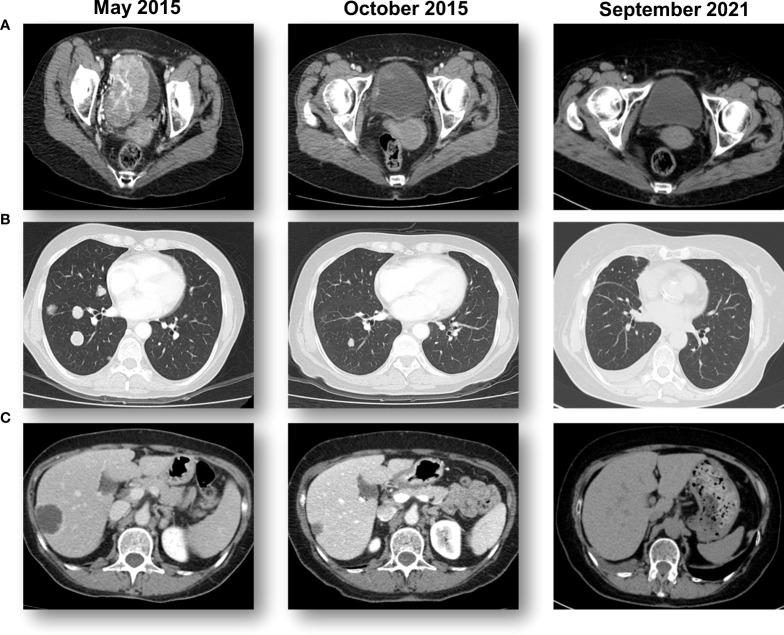
Sequential CT scans showing a response to pemprolizumab. **(A)** A large bladder tumor that was biopsied prior to starting pembrolizumab immunotherapy with a complete response in the bladder. **(B)** Multiple lung metastases from bladder cancer that obtained a complete response with pembrolizumab immunotherapy. **(C)** Multiple hepatic metastases from bladder cancer that obtained a complete response with pembrolizumab immunotherapy.

A 61-year-old woman presented with hematuria in July 2014. In November 2014, computed tomography (CT) scans and a transurethral resection of bladder tumor (TURBT) revealed the presence of a G3T1 bladder tumor. A second CT scan in January 2015 showed a large bladder tumor and metastases in the lung and liver (**Figure 1A**; **Supplementary Figure S1**). Another TURBT in January 2015 again revealed a G3T1 bladder tumor. After undergoing five cycles of GC and cisplatin chemotherapy as first-line treatment, CT scans in May 2015 revealed progressive disease (PD) in the liver, lung, and bladder. She was offered standard-of-care treatment, vinflunine, in her city, but elected to participate in an ongoing clinical study in Rome, Italy (**Supplementary Figure S1**).

The patient participated in the KEYNOTE-045 study, an international, randomized, and open-label phase III study, in which a total of 542 patients were enrolled and randomized between pembrolizumab and the physician’s choice of chemotherapy (docetaxel, paclitaxel, or vinflunine). The patient was randomized to receive pembrolizumab 200mg (q3w) starting on June 30, 2015. Sequential CT scans revealed a rapid reduction of the tumors in all three regions (**Figure 1**).

**Figure 2:**
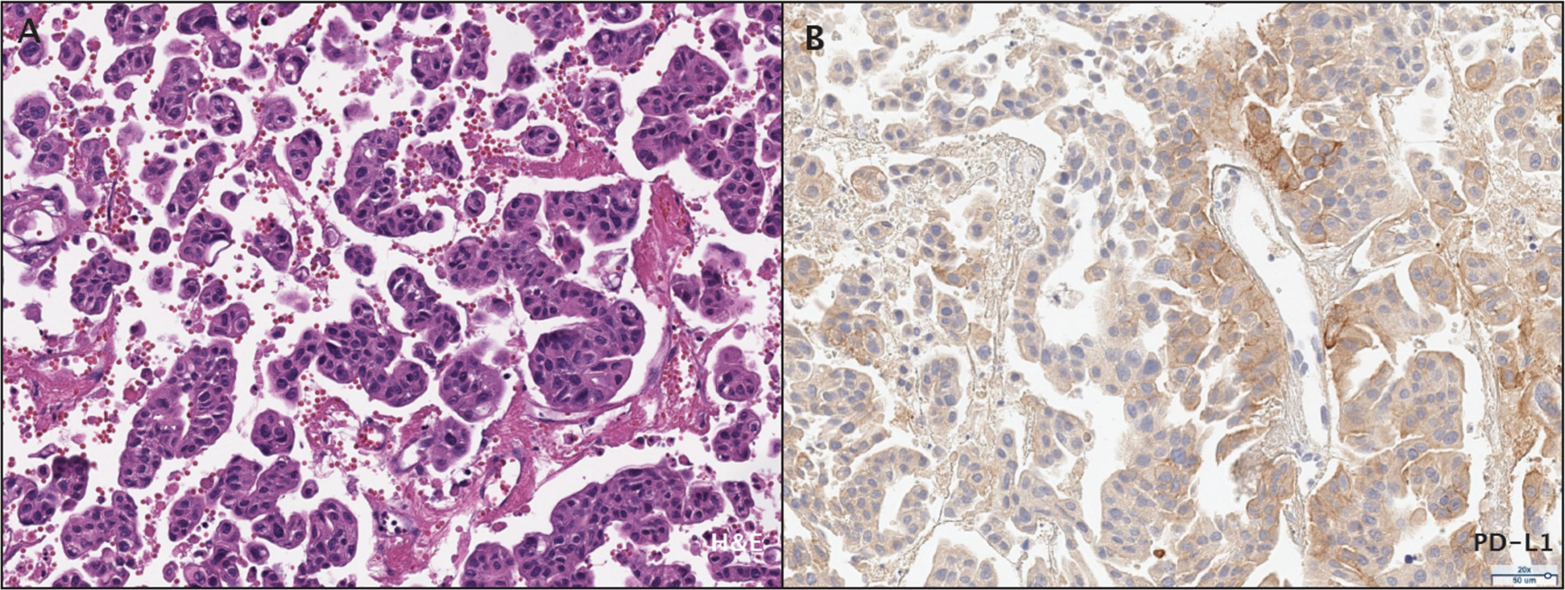
Histopathological assessment of the patient's bladder urothelial carcinoma. **(A)** Micropapillary histopathology was observed by H&E staining. **(B)** PD-L1 expression was assessed by immunohistochemistry.

Histologically, a micropapillary variant was observed, which is rare (0.6 - 8.2%) and has been shown to have poor prognosis in UC (10). Immunohistochemistry (IHC) demonstrated 15% of PD-L1 expression (DAKO 22C3) (**Figure 2**). The patient responded dramatically and continued pembrolizumab treatment for 2 years per the protocol. Serial CT scans showed that she has been free of all disease and off of therapy for over 6 years (**Figure 1**; **Supplementary Figure S1**).

**Figure 3:**
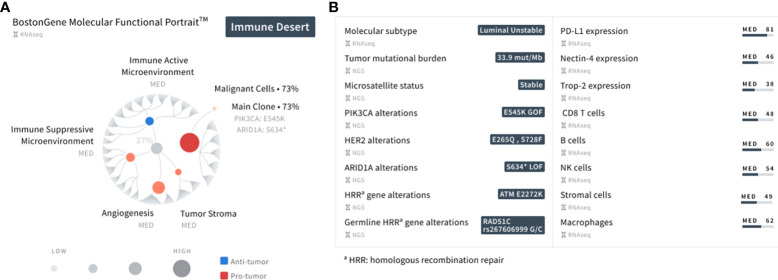
Summary of the patient’s major findings using the MF Portrait^9^ and integrative genomic and transcriptomic analysis (Data Supplement). **(A)** A planetary schematic representation of the molecular and functional characteristics of the patient’s tumor. Blue represents anti-tumor activity (immune active microenvironment) and red represents pro-tumor activity (immune suppressive microenvironment, angiogenesis, tumor stroma). The size of the dot corresponds to the strength of the presented gene signatures. **(B)** List of key findings using WES and RNA-seq analysis represented as NGS and RNA-seq, respectively.

The BostonGene Tumor Portrait™ Test, a comprehensive genomic and transcriptomic analysis using whole-exome sequencing (WES) and RNA sequencing (RNA-seq), was performed to understand the mechanisms of response to immunotherapy. Based on transcriptomic profiling and the TME subtype represented as the MF Portrait in this test, the patient’s tumor can be described as an “Immune Desert” (9), which is usually characterized by a non-inflamed TME and a high tumor proliferation rate (**Figure 3**). All components of the patient’s tumor, including malignant cells, tumor stroma, angiogenesis, and an immune active and suppressive microenvironment were ranked at a medium level (Data Supplement) in comparison to the cohort with a similar diagnosis, as well as T cells, B cells, NK cells, neutrophils, monocytes (including macrophages), and stromal cells. The tumor purity was 73%. The patient’s tumor was characterized by a high TMB level (33.9 mut/mb), which corresponds with a good response to immunotherapy (11). APOBEC-related mutagenesis is a major source of mutations in bladder cancer (12); therefore, the presence of a large number of mutations may be due to the activation of the APOBEC complex, which was confirmed by the presence of a pronounced mutational signature. The tumor’s microsatellite status was stable.

Further characterization of the tumor showed the onset of at least one clone with a *PIK3CA* gain of function *E545K* missense mutation, *ARID1A* loss of function S634* nonsense mutation, and a *HER2 E265Q* (VAF 26%) and *S728F* (VAF 25%) missense mutation. It has been previously documented that micropapillary UC exhibits a high frequency of *HER2* activating mutations (13). The *ATM* missense mutation E2272K was also observed in the TME. Mutations in *PIK3CA*, *ARID1A*, and *ATM* are recurrent genetic alterations in bladder cancer (14), possessing the ability to influence response to several treatments, such as ICIs (15–17). The *RAD51C* mutation was observed along with the ATM mutation in homologous recombination genes. A summary of the patient’s findings is shown in **Figure 3**. Informed consent was obtained from the patient in this report for the collection and publication of her clinical and molecular data, and de-identified images.

## Discussion

Herein, we report the case of a patient with metastatic UC who exhibited an exceptional response to pembrolizumab, and subsequently describe the use of the MF Portrait to characterize the tumor and TME. Several ICIs have been provisionally FDA-approved as second-line therapies after platinum failure based on phase I-III trials, with treatment responses around 20%. While pembrolizumab is approved in patients who are platinum ineligible and PD-L1 positive and in patients with non-muscle invasive bladder cancer with carcinoma *in situ* who have failed *Bacille Calmette-Guérin* (BCG) treatment, atezolizumab and durvalumab have withdrawn their indication after large phase III trials in combination with immunotherapy, or compared to immunotherapy, failed to meet their primary endpoints (18, 19). The KEYNOTE-045 study was the only phase III randomized trial in this setting that enrolled patients with predominantly transitional cell UC of the renal pelvis, ureter, bladder, or urethra, with progressive disease after receiving two or fewer lines of platinum-based chemotherapy or with recurrence less than 12 months after perioperative platinum-based chemotherapy. Updates of the KEYNOTE-045 study have consistently shown the benefit of pembrolizumab in the second-line setting, irrespective of PD-L1 status (4, 20).

The patient described here exhibited a rapid, long-lasting exceptional response to pembrolizumab immunotherapy, since 2015 and remains in CR, to our knowledge as of May 2022. Integrated genomic and transcriptomic analysis was utilized to understand the characteristics of the tumor and investigate its possible associations with the patient’s response (Data Supplement). The MF Portrait classified the patient’s tumor as an Immune Desert, and further analysis detected a high TMB and APOBEC signature in the patient’s bladder tumor sample. According to the most recent UC classifier described by Kamoun et al. (21), the patient’s molecular profile is categorized as a Luminal Unstable subtype, which is distinguished by an APOBEC signature and a high TMB. Patients classified as Luminal Unstable have a good prognosis, as the survival rate of this subtype is better than in other subtypes, with the exception of the Luminal Papillary subtype (21).

Positive biomarkers for ICI exist, such as high TMB and the presence of mutations in *ARID1A, ATM*, and *PIK3CA*. Therefore, connecting the tumor characteristics with the patient’s response may enable therapeutic decision-making for other patients who exhibit similar features. While sequencing technologies were not available before or during therapy for the patient presented here, these results suggest that performing comprehensive molecular profiling before first-line treatment can lead to the discovery and establishment of biomarker-driven therapies for UC patients in the future. Furthermore, this case further demonstrates the utility of performing integrated genomic and transcriptomic analysis before second-line ICI treatment to identify biomarkers that can help stratify patients for immunotherapy or clinical trial enrollment. For instance, particularly in this case, detection of the APOBEC signature suggests that the mutation might serve as a predictive biomarker for better response to ICI (22). Similarly, *ATM* and *ARID1A* mutations might also be associated with a higher mutational load and better response to immunotherapy (15). Of note, the *ATM* mutation can also indicate the use of PARP inhibitors such as olaparib (NCT03375307), neratinib (NCT03830918), and rucaparib in other tumor types. ATM is one of the DNA damage response mechanism regulators, and tumor cells cannot repair DNA damage in the absence of PARP proteins (23). *E545K*, which is commonly found in UC, is a gain of function missense mutation in the helical domain of PIK3CA (24). Knockdown of the PIK3CA mutant variant inhibits cell proliferation and migration, and hence, tumor growth (25). Consequently, it is appropriate to use PIK3CA inhibitors alone or in combination (NCT04317105, NCT02465060) when the E545K mutation is detected.

Importantly, response to ICI does not prevent the acquisition of multiple resistance mechanisms (26), including TME-driven resistance (27). Therefore, combination strategies that increase ICI efficacy are of great interest to clinicians and scientists (28). Here, another advantage of the MF Portrait is revealed. Particularly, characterization of the tumor and TME using the MF Portrait provides easy access to the proportion of potentially actionable TME components, such as endothelial, stromal, and immune active/suppressive cells. Our results suggest that the MF Portrait has the potential to be used as a predictive biomarker for UC. Future prospective clinical studies will be critical to further validate the predictive ability of the MF Portrait for response to immunotherapy.

## Conclusions

This paper describes a patient with micropapillary advanced UC with bladder, liver, and lung metastases. After failing cisplatin-based combination chemotherapy, the patient was treated with pembrolizumab immunotherapy on the KEYNOTE-045 randomized Phase III trial for two years. The patient is currently disease free and has been off all therapy for over 6 years. Her case is that of an exceptional responder. The patient’s molecular profile is consistent with her response to immunotherapy and we have performed tumor profiling to further describe her molecular characteristics.

## Materials and methods

### Tumor tissue collection

A transurethral resection of bladder tumor (TURBT) was performed on a 61-year-old female in 2015 in Milan, Italy before first-line cisplatin-based chemotherapy treatment. Tumor tissue from the surgery was used for histological assessment and molecular profiling at Weill Cornell Medicine in New York City, New York and at BostonGene in Waltham, Massachusetts. No other metastatic biopsies were obtained prior to pembrolizumab.

### Histopathological assessment

Hematoxylin and eosin-stained (H&E) slides were reviewed at Weill Cornell Medicine by a pathologist with expertise in uropathology. The combined positive score (CPS) was determined to measure immunohistochemistry of PD-L1 expression in both tumor and inflammatory cells.

### Molecular and bioinformatics analysis

DNA and RNA of the tumor were extracted from formalin-fixed paraffin-embedded (FFPE) tissue using the FFPE AllPrep DNA/RNA kit (Quiagen). Normal DNA was extracted from blood using the QIAamp DNA Blood Mini Kit (Qiagen).Library preparation from extracted DNA was performed with the Agilent SureSelect XT HS2 DNA kit for library construction and the Agilent SureSelect Human All Exon V7 exome for hybridization and capture. Library preparation from RNA was performed with the Agilent SureSelect XT HS2 RNA kit for library construction and the Agilent SureSelect Human All Exon V7+UTR exome for hybridization and capture. RNA-seq and whole exome sequencing (WES) were performed on an Illumina NovaSeq 6000 system. FastQC v0.11.9, FastQ Screen v0.14.0, and MultiQC v1.4 were used to conduct next-generation sequencing quality control analysis.

### Genomic alterations

Low-quality reads for WES were filtered with FilterByTile/BBMap v37.90 and then aligned to the human reference genome GRCh38 (GRCh38.d1.vd1 assembly) using BWA v0.7.17. Picard’s (v2.6.0) Mark Duplicates was used to remove duplicate reads. Indels were realigned by IndelRealigner and recalibrated using BaseRecalibrator (GATK v4.1.2.0). Strelka v2.9.10 and Variant Effect Predictor v92.1 were used to detect and annotate, respectively, germline and somatic single-nucleotide variations, small insertions, and deletions. CNA evaluation was performed by a customized version of Sequenza v2.1.2.

Microsatellite status was evaluated by MSI sensor v0.6.

### Expression analysis

RNA-seq reads were processed using a custom BostonGene pipeline. Briefly, reads were aligned using Kallisto v0.43.0 to GENCODE v23 transcripts 69. Noncoding, histone- and mitochondrial-related transcripts were removed, and protein coding, IGH/K/L- and TCR-related transcripts were retained, resulting in 20,062 analyzed genes. Gene expression was quantified as transcripts per million (TPM) and log2 transformed.

Expression biomarkers were calculated based on RNA-seq data from BostonGene’s internal diagnosis-stratified patient cohort. Gene expression levels (high, medium, or low) were calculated and shown relative to their expression level in patients with a similar diagnosis (“low” corresponds to expressions below the 17th percentile, “high” corresponds to above the 83rd percentile, and what remains corresponds to “medium”). The cut-off value for positive expression was defined as 1TPM.

Fusions were detected using STAR-Fusion v.1.8.1.

### TME processes activity estimation

To estimate the activity of specific genes we used methodology described by Bagaev et. al (9). Using functional gene expression signatures (Fges), we analyzed genes associated with distinct cell types (e.g., macrophages, tumor infiltrating lymphocytes), non-cellular components of the TME (e.g., immunosuppressive cytokines, extracellular matrix), malignant cell biological processes (e.g., proliferation), and canonical signaling pathway activation (e.g., TGFb, TP53). The ssGSEA algorithm was used to estimate Fges activity (29). The Fges activity level (high, medium, or low) was calculated and shown relative to its Fges activity level in patients with similar diagnosis (“low” corresponds to Fges scores below the 17th percentile; “high” corresponds to scores above the 83rd percentile; and the remainder corresponds to “medium”).

## Data availability statement

The original contributions presented in the study are included in the article/**Supplementary Material**, further inquiries can be directed to the corresponding author/s.

## Ethics statement

The studies involving human participants were reviewed and approved by Weill Cornell Medicine IRB. The patients/participants provided their written informed consent to participate in this study. Written informed consent was obtained from the individual(s) for the publication of any potentially identifiable images or data included in this article.

## Author contributions

CS and JMM contributed equally to this work and share senior authorship. CS and JMM conceived of the paper. The patient was treated by CS, FC, LC, and GP. KC, NM, GS, AZ, and NaiS conducted the overall data analyses and sequencing data processing. JMM and MA performed histological assessment of the tissue slides. CS, NarS, KC, AZ, AB, JB, LY, DG, and JMM wrote and revised the manuscript and prepared the Figures. All authors contributed to the article and approved the submitted versions.

## Funding

The Keynote study was funded by Merck, Sharp and Dohme, LLC. and data analysis was funded by BostonGene. The study was supported by the Caryl and Israel Englander Institute for Precision Medicine at Weill Cornell Medicine. 

## Conflict of interest

Authors NaiS, KC, NM, GS, AZ, NarS, AB, JB and LY were employed by BostonGene, Corp. CNS has received honoraria from MERCK and participated as an author in the Keynote-045 trial.

The remaining authors declare that the research was conducted in the absence of any commercial or financial relationships that could be construed as a potential conflict of interest.

The authors declare that this study received funding from BostonGene, Merck and Sharp and Dohme, LLC. Corp. BostonGene had the following involvement with the study: analysis, interpretation of data, and the writing of this article. Merck, Sharp and Dohme, LLC had the following involvement with the study: study design of Keynote 045, sample collection of tissue from San Camillo Hospital, and the decision to submit this article for publication.

## Publisher’s note

All claims expressed in this article are solely those of the authors and do not necessarily represent those of their affiliated organizations, or those of the publisher, the editors and the reviewers. Any product that may be evaluated in this article, or claim that may be made by its manufacturer, is not guaranteed or endorsed by the publisher.
