# Supplementary figures and images for: Case report: Metastatic urothelial cancer with an exceptional response to immunotherapy and comprehensive understanding of the tumor and the tumor microenvironment
Source: Front Oncol. 2022 Oct 31;12:1006017. doi: 10.3389/fonc.2022.1006017 (PMC9661726; doi:10.3389/fonc.2022.1006017)

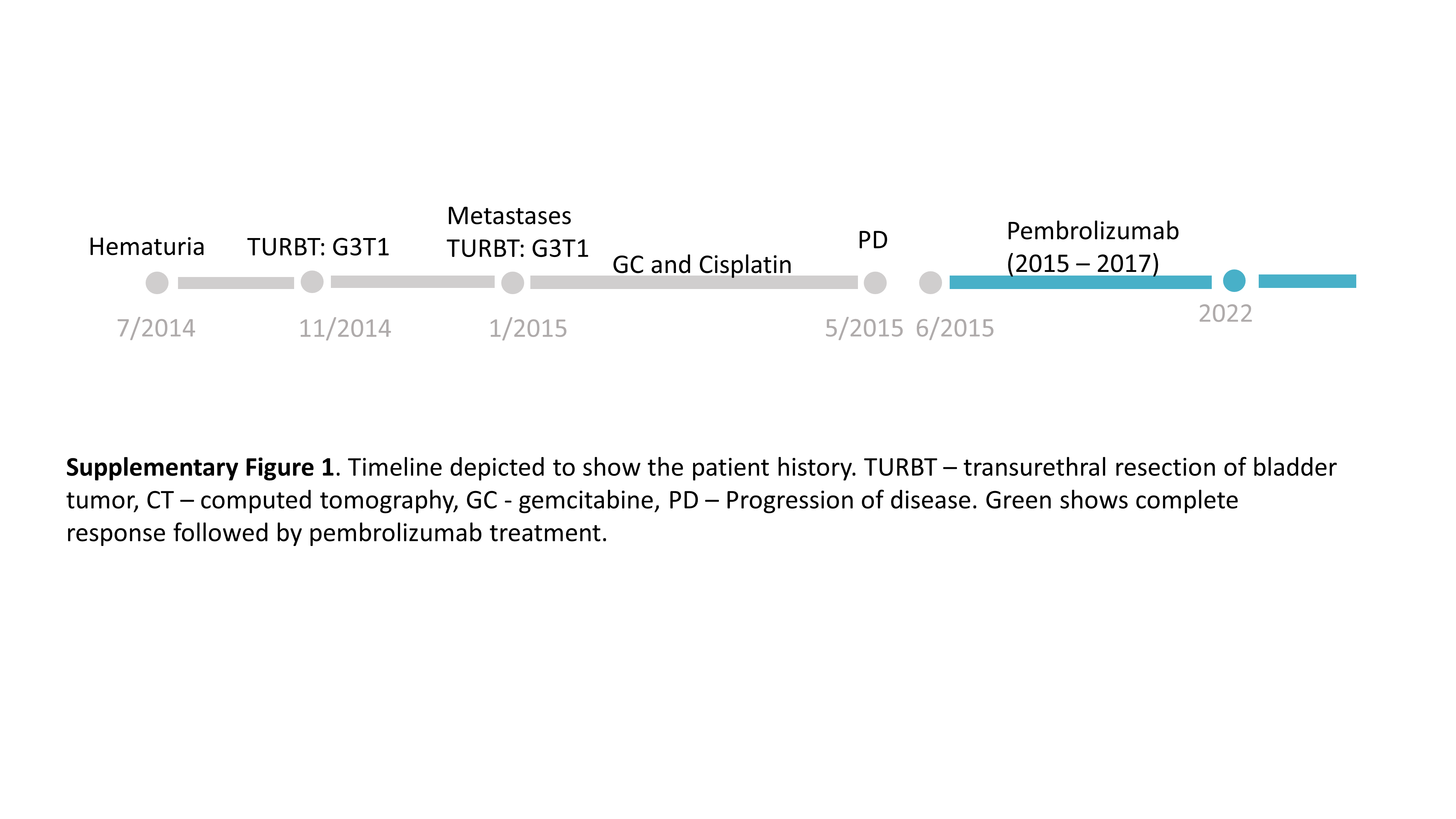

Supplement: Supplementary file 1 [file Image_1.tif]
